# A mixed‐methods study of cyclin‐dependent kinase 4 and 6 inhibitor symptom burden and quality of life among metastatic breast cancer patients and providers

**DOI:** 10.1002/cam4.4055

**Published:** 2021-06-24

**Authors:** Laura B. Oswald, Brandy Arredondo, Mika Kadono, Dinorah Martinez‐Tyson, Cathy D. Meade, Frank Penedo, Michael H. Antoni, Hatem Soliman, Ricardo L. B. Costa, Heather S. L. Jim

**Affiliations:** ^1^ Health Outcomes and Behavior Program Moffitt Cancer Center Tampa FL USA; ^2^ Participant Research, Interventions, and Measurement Core Moffitt Cancer Center Tampa FL USA; ^3^ Institute for Health Equity AltaMed Health Services Los Angeles CA USA; ^4^ College of Public Health University of South Florida Tampa FL USA; ^5^ Department of Psychology University of Miami Coral Gables FL USA; ^6^ Department of Medicine University of Miami Coral Gables FL USA; ^7^ Breast Oncology Program Moffitt Cancer Center Tampa FL USA

**Keywords:** breast neoplasms, neoplasm metastasis, patient‐reported outcomes measures, psycho‐oncology, qualitative research, quality of life

## Abstract

**Background:**

Cyclin‐dependent kinase 4 and 6 (CDK4/6) inhibitor targeted therapies dramatically improve survival outcomes for metastatic breast cancer (MBC), but they are associated with significant symptom burden that can impact patients’ health‐related quality of life (HRQOL) and treatment outcomes. This study is the first to describe CDK4/6 inhibitor symptoms from the lived perspectives of MBC patients taking CDK4/6 inhibitors and healthcare providers involved in MBC care. This study also explored patients’ symptom management and HRQOL concerns, and gathered feedback about developing supportive interventions for MBC.

**Methods:**

MBC patients taking CDK4/6 inhibitors (*N* = 20) and MBC healthcare providers (*N* = 12) participated in semi‐structured interviews that were analyzed for qualitative themes. MBC patients completed surveys about HRQOL, symptoms, and unmet needs.

**Results:**

Patient and provider perceptions of CDK4/6 inhibitor symptoms did not align with patients perceiving symptoms as more burdensome. Patients reported that supportive resources (e.g., support groups, blogs) that are not specific to MBC do not adequately meet their needs. Patients and providers were enthusiastic about developing supportive interventions specifically for MBC and offered considerations for designing such interventions.

**Conclusions:**

Findings highlight differences in perceptions of CDK4/6 inhibitor symptom burden between MBC patients and providers. Results will inform the development of supportive interventions to assist MBC patients in managing CDK4/6 inhibitor symptom burden and maintaining HRQOL. Such interventions could also improve treatment outcomes.

## Abstract 2

Metastatic breast cancer (MBC) patient and provider perceptions of symptoms associated with CDK4/6 inhibitor did not align, with patients perceiving symptoms as more burdensome. Patients reported that supportive resources (e.g., support groups, blogs) that are not specific to MBC do not adequately meet their needs, however patients and providers were enthusiastic about developing supportive interventions specifically for MBC and offered considerations for designing such interventions. Results will inform the development of supportive interventions to assist MBC patients in managing CDK4/6 inhibitor symptom burden and maintaining health‐related quality of life.

## 1 INTRODUCTION

Over the past several decades, the population of metastatic breast cancer (MBC) patients in the United States has grown steadily,^1^ and the vast majority of MBC patients (>70%) have hormone receptor‐positive (HR+) and human epithelial growth factor 2 negative (HER2−) disease.^2^ Patients’ responses to a MBC diagnosis are multifaceted and often characterized by uncertainty about prognosis and survival, lack of control, and significant emotional distress.^3^, ^4^, ^5^ Recently, treatment for HR+/HER2− MBC has been revolutionized by cyclin‐dependent kinase 4 and 6 (CDK4/6) inhibitor targeted therapies. Across clinical trials, treatment with CDK4/6 inhibitors plus endocrine therapy led to double the progression‐free survival relative to endocrine therapy alone (median almost 2 years vs. <1).^6^, ^7^, ^8^, ^9^, ^10^, ^11^, ^12^ Thus, CDK4/6 inhibitors offer renewed hope to MBC patients.

Despite their clinical efficacy, CDK4/6 inhibitors are associated with significant symptom burden that can limit tolerability. In clinical trials, up to 89% of patients experienced a Grade 3–4 adverse event (AE).^13^ While many AEs were asymptomatic (e.g., hematological AEs), other prevalent symptoms may impact patients’ health‐related quality of life (HRQOL) (e.g., fatigue, diarrhea).^14^ One clinical trial documented a clinically meaningful HRQOL deterioration in a quarter of patients over 1 year.^15^ As a result, more than 70% of clinical trial patients had their treatment dose reduced and more than 15% discontinued treatment altogether.^13^ Symptom burden could also impact adherence, as research shows greater symptom burden is associated with worse adherence to oral cancer treatments.^16^ These outcomes are critically important, because MBC patients must remain on treatment indefinitely in order to keep their disease controlled. Thus, managing symptoms and addressing associated HRQOL concerns are essential for keeping MBC patients on life‐saving treatments and providing high‐quality care.^5^, ^17^

There is strong evidence that supportive interventions using evidence‐based approaches (e.g., cognitive‐behavioral theory) can improve HRQOL and symptom outcomes in breast cancer.^18^, ^19^, ^20^, ^21^ However, supportive interventions that are specifically targeted to MBC concerns are scarce.^22^, ^23^, ^24^ When developing supportive interventions for MBC, it is critical to consider patients’ lived experiences on treatment to ensure that resulting interventions accurately capture and address their concerns. Yet to date, little is known about MBC patients’ lived experiences with CDK4/6 inhibitor targeted therapies.

To address this gap in knowledge, the purpose of the study was to describe symptoms associated with CDK4/6 inhibitors from the lived perspectives of MBC patients as well as healthcare providers involved in MBC care via semi‐structured interviews (with patients and providers) and surveys (completed by patients only). This study also sought to explore MBC patients’ HRQOL concerns and gather feedback about developing supportive interventions to improve these outcomes.

## 2 METHODS

### 2.1 Participants

Eligible patients were: (i) female; (ii) ≥18 years old; (iii) diagnosed with HR+/HER2− MBC; (iv) prescribed a CDK4/6 inhibitor (i.e., palbociclib, ribociclib, abemaciclib) for ≥4 weeks; (v) expected to survive ≥3 months; (vi) able to speak and read English. Patients with documented/observable psychiatric or neurological disorders that could interfere with participation were excluded (e.g., dementia, psychosis). Eligible providers were healthcare professionals who provided MBC care (e.g., MDs, APRNs).

### 2.2 Procedures

This study was reviewed by the Advarra Institutional Review Board and deemed exempt from oversight due to minimal risk (Pro0004135). From April to May 2020, a study coordinator worked with staff in Moffitt Cancer Center’s Breast Oncology Clinic to identify eligible patients. After verbal consent, patients completed individual telephone interviews lasting approximately one hour and an online survey assessing HRQOL, symptoms, and unmet needs. Patients received $50 for participating. Providers were recruited by emailing medical oncologists in Moffitt Cancer Center’s Breast Oncology Clinic, and oncologists nominated other treatment team members for participation. After verbal consent, providers completed individual telephone interviews lasting approximately one hour. Providers were not compensated. Patients and providers were recruited and interviewed continuously until thematic saturation was reached (i.e., no new themes emerged during subsequent interviews). Past work has shown that thematic saturation occurs within 12 individual interviews, with the most important elements of meta‐themes evident after as few as six individual interviews.^25^

### 2.3 Semi‐structured interviews

Trained study team members conducted interviews using semi‐structured guides containing a list of questions and exploratory probes. Patient interviews focused on their MBC‐related experiences, CDK4/6 inhibitor symptoms (e.g., prevalence, frequency, impact), psychosocial concerns (e.g., relationship changes, existential concerns), and interest in supportive interventions to address these concerns. Provider interviews focused on their experiences providing MBC care, common CDK4/6 inhibitor symptoms, and additional MBC‐related concerns. To facilitate discussions about symptoms, participants were shown the patient‐reported outcomes version of the Common Terminology Criteria for Adverse Events (PRO‐CTCAE) item library of 78 patient‐reported AEs derived from the CTCAE.^26^, ^27^

### 2.4 Patient surveys

#### 2.4.1 HRQOL

The 37‐item Functional Assessment of Cancer Therapy‐Breast (FACT‐B) assessed physical, social, emotional, and functional well‐being and additional breast cancer concerns.^28^, ^29^ Patients indicated how true statements were in the past week on a Likert‐type scale from 0 (not at all) to 4 (very much). Physical, social, emotional, and functional well‐being items were summed to indicate general HRQOL (FACT‐G total, possible range 0–108). Items related to additional breast cancer concerns were added to the FACT‐G total score to indicate breast cancer HRQOL (FACT‐B total, possible range 0–148). Higher scores indicated better HRQOL.

#### 2.4.2 Symptom burden

The 18‐item Breast Cancer Prevention Trial (BCPT) Symptom Scales assessed eight symptom clusters: hot flashes, nausea, bladder control, vaginal problems, musculoskeletal pain, cognitive problems, weight problems, and arm problems.^30^ Patients indicated how bothered they were by symptoms in the past 4 weeks on a Likert‐type scale from 0 (not at all) to 4 (extremely). Items within symptom clusters were averaged to produce cluster scores. All items were averaged to indicate total symptom burden. Higher scores indicated more/worse symptom burden.

#### 2.4.3 Fatigue

Fatigue is a hallmark symptom of CDK4/6 inhibitors not captured by the BCPT Symptom Scales. On the 13‐item Functional Assessment of Chronic Illness Therapy (FACIT)‐Fatigue, patients indicated how true fatigue‐related statements were in the past week on a Likert‐type scale from 0 (not at all) to 4 (very much).^31^, ^32^ Items were summed and lower scores indicated worse fatigue (possible range 0–52). A cutoff of ≤34 indicated severe fatigue.^33^

#### 2.4.4 Unmet needs

The 23‐item Needs Evaluation Questionnaire (NEQ) assessed the incidence of unmet needs related to: information, assistance/care, social, psychological/emotional, and material/economic needs.^34^, ^35^ Items were summed to produce domain scores and a total unmet needs score (possible range 0–23). Higher scores indicated more unmet needs.

### 2.5 Analyses

After reaching thematic saturation in the semi‐structured interviews, descriptive statistics were used to characterize patients’ demographics, clinical characteristics, and survey data as well as providers’ demographics and credentials. The audio‐recorded interviews were transcribed verbatim and analyzed for qualitative themes using NVivo 11 software according to NIH best practices for mixed methods research.^36^ Two team members independently reviewed the transcripts and created a codebook using a priori themes derived from the interview guides. The coders achieved high intercoder reliability (κ = 0.84), iteratively coded the interviews, added emergent codes throughout the coding process, identified major themes, and extracted representative quotes. Coding discrepancies were resolved by consensus. The data that support the findings of this study are available from the corresponding author upon reasonable request.

## 3 RESULTS

### 3.1 Participant characteristics

The sample included 20 patients and 12 providers. Table 1 describes patients’ demographics and clinical characteristics and providers’ demographics and credentials. On average, patients were of 59 years old (standard deviation [SD] = 12) and mostly White (90%) and non‐Hispanic/Latina (95%). On average, patients had been diagnosed with breast cancer 4 years earlier (SD = 6) and were taking a CDK4/6 inhibitor for 15 months (SD = 10). Most patients were prescribed palbociclib (70%). Providers were medical oncologists (67%), advanced practice nurse practitioners (17%), and physician assistants (17%). On average, providers had 10 years of experience working in oncology post‐training (SD = 10) and had provided MBC care for 10 years (SD = 11).

**TABLE 1:** Metastatic breast cancer patients’ demographics and clinical characteristics (*N* = 20) and providers’ demographics and credentials (*N* = 12)

	Statistic
Patient characteristics	
Age; *M* (SD) range	59.0 (12.3) 40–79
Race; *n* (%)	
White	18 (90.0)
Black or African American	1 (5.0)
More than one race	1 (5.0)
Ethnicity; *n* (%)	
Hispanic/Latina	1 (5.0)
Non‐Hispanic/Latina	19 (95.0)
Marital status; *n* (%)	
Married	11 (55.0)
Divorced	4 (20.0)
Widowed	3 (15.0)
Never married	2 (10.0)
Highest level of education completed; *n* (%)	
High school	2 (10.0)
Some college	8 (40.0)
College	6 (30.0)
Graduate degree	4 (20.0)
Annual household income; *n* (%)	
$10,000–$19,999	5 (25.0)
$20,000–$39,999	3 (15.0)
$40,000–$59,999	—
$60,000–$99,999	5 (25.0)
$100,000 or more	4 (20.0)
Prefer not to answer	3 (15.0)
Years since breast cancer diagnosis; *M* (SD) range	4.3 (6.4) 0–20
Prescribed CDK4/6 inhibitor; *n* (%)	
Palbociclib	14 (70.0)
Ribociclib	3 (15.0)
Abemaciclib	3 (15.0)
Months taking CDK4/6 inhibitor; *M* (SD) range	15.4 (10.2) 1–30
Provider characteristics	
Gender; *n* (%)	
Male	3 (25.0)
Female	9 (75.0)
Race; n (%)	
White	9 (75.0)
Asian	2 (16.7)
More than one race	1 (8.3)
Ethnicity; *n* (%)	
Hispanic/Latino	2 (16.7)
Non‐Hispanic/Latino	10 (83.3)
Highest degree; *n* (%)	
Doctor of Medicine (MD)	8 (66.7)
Advanced Practice Nurse Practitioner (APRN)	2 (16.7)
Physician Assistant‐Certified (PA‐C)	2 (16.7)
Years since earning highest degree; *M* (SD) range	14.2 (12.7) 3.0–46.0

Abbreviations: CDK4/6, cyclin‐dependent kinase 4 and 6; SD, standard deviation.

### 3.2 Survey results

Table 2 describes patients’ survey responses. On the FACT‐B, the average breast cancer HRQOL score was 104.9 (SD = 18.4) and the average general HRQOL score was 80.3 (SD = 12.5). On the BCPT Symptom Scales, average total symptom burden was slightly to moderately bothersome (*M* = 1.3, SD = 0.7). Musculoskeletal pain was the most bothersome symptom cluster, with each symptom rated at least moderate. On the FACIT‐Fatigue, the average total fatigue score exceeded the severity cutoff (*M* = 33.8, SD = 12.1). On the NEQ, patients reported an average of 2.8 unmet needs (SD = 3.5). Most patients reported 1–4 unmet needs (55%) and fewer reported 0 (30%) or >4 (15%). The most frequently endorsed needs were information about the future condition (endorsed by 45%), better symptom control (40%), economic help (30%), more involvement in treatment choices (25%), economic/insurance information (25%), need to speak with peers (20%), treatment information (20%), and easier‐to‐understand information (20%).

**TABLE 2:** Metastatic breast cancer patients’ survey data

	*M* (SD) Range
HRQOL	
General HRQOL (FACT‐G total)	80.3 (12.5) 57.0–103.0
Breast cancer HRQOL (FACT‐B total)	104.9 (18.4) 69.0–134.0
Symptom burden	
Total symptom burden	1.3 (0.7) 0.1–2.2
Hot flashes	1.3 (1.3) 0.0–3.5
Nausea	0.2 (0.3) 0.0–1.0
Bladder control	0.6 (0.7) 0.0–2.5
Vaginal problems	1.6 (1.6) 0.0–4.0
Musculoskeletal pain	2.2 (1.1) 0.3–4.0
Cognitive problems	1.6 (1.4) 0.0–3.7
Weight problems	1.6 (1.2) 0.0–3.5
Arm problems	0.7 (1.1) 0.0–3.0
Fatigue*	
Total fatigue	33.8 (12.1) 13.0–50.0
Unmet needs	
Total unmet needs	2.8 (3.5) 0.0–11.0
Information needs	1.6 (2.2) 0.0–7.0
Assistance/care needs	—
Social needs	0.3 (0.7) 0.0–2.0
Psychological and emotional needs	0.3 (0.6) 0.0–2.0
Material/economic needs	0.6 (0.8) 0.0–2.0

Abbreviations: FACT‐B, Functional Assessment of Cancer Therapy‐Breast; FACT‐G, Functional Assessment of Cancer Therapy‐General; HRQOL, health‐related quality of life; SD, standard deviation.

* Scores ≤34 indicate severe fatigue.

### 3.3 Qualitative themes

Three qualitative themes emerged: (i) perceptions of CDK4/6 inhibitor symptom burden did not align; (ii) patients are perceived to have good HRQOL; and (iii) supportive resources not specific to MBC are inadequate. Additional representative quotes for each theme are included in Table S1.

#### 3.3.1 Perceptions of symptom burden did not align

Patients described a host of CDK4/6 inhibitor symptoms that affected their lives in many ways. Some symptoms were more common but less burdensome (e.g., nausea), whereas others were less common but very burdensome (e.g., sexual dysfunction). Fatigue was both prevalent and highly burdensome. One patient explained:

“I had slight fatigue with the cancer diagnosis. But… I noticed an increase in fatigue [with the CDK4/6 inhibitor] to the point where I have a lot of trouble driving… I will sleep a total of three to four hours during my day.” (PT016)

Providers, however, perceived CDK4/6 inhibitor symptoms as minimal and tolerable. Some providers expressed their perceptions of tolerability specifically within the context of treating incurable cancer, suggesting that tolerating symptoms is necessary for keeping patients on treatment. Most patients reported that they disclosed their symptoms to their medical teams. Those that did not offered a variety of reasons for symptom nondisclosure (e.g., symptoms were expected, symptoms were not concerning enough for the patient or provider, embarrassment about symptoms and/or asking questions). This is shown in the following patient quote:

“I kind of feel a little stupid because I ask the oncologist about some things and he says, ‘Well, you need to ask your primary about that.’ So, then I asked, ‘Well, who do I call if I get sick?’ I don't know how any of this works, really.” (PT009)

#### 3.3.2 Patients are perceived as having good HRQOL

Patients reported good overall HRQOL and often contextualized their perspectives with regard to their prognosis. Many patients shared that MBC led to changes in their work status, which affected their social lives, identity, financial security, and relationships. Several patients explicitly acknowledged that their cancer was incurable. This acceptance altered their life outlooks, future planning, and expectations of physical activities. This also led many patients to cultivate deeper connections with family and friends. Providers also viewed patients as having good HRQOL and acknowledged that maintaining HRQOL is a priority in MBC clinical care in order to facilitate a consistent course of treatment. Some providers described having to delicately balance patients’ treatment dose and HRQOL with patients’ anxiety about dose reductions to alleviate symptoms. One provider explained:

“I often hear, ‘Well, let me just stick it out a little longer.’ And so often it takes a few months before I can actually reduce [the treatment dose to alleviate] physical symptoms… So, in some ways I see [patients] choosing to persevere over quality of life concerns. And that does concern me… [It’s a] marathon not a sprint and beating up our bodies repeatedly month after month is not necessarily a good approach, because they might get exhausted.” (PV512)

#### 3.3.3 Supportive resources not specific to MBC are inadequate

Patients expressed a desire for supportive resources (e.g., support groups, blogs) that are specific to MBC concerns, positive in tone, and informational. They reported finding limited utility in resources not designed for MBC patients, as they viewed their experiences with having incurable metastatic disease as very different than the experiences of cancer patients with potentially curable disease (e.g., differing prognosis or treatment regimens). For example, one patient described:

“[W]hen I speak to other cancer survivors, I’m not the same as them. I talked to them. They always speak to me in regard to surgery and intravenous chemotherapy, and their experience is completely different from mine. And it’s hard to relate [to] those aspects.” (PT020)

Patients identified potential benefits of engaging with MBC‐specific supportive resources, such as being able to learn about their treatments, symptoms, and ways to manage stress. When delivered in a group setting, patients viewed MBC‐specific supportive interventions as spaces to share their stories, learn from others’ experiences, and connect with their peers. Patients expressed particular interest in peer‐mentoring, but they feared hearing “horror stories” and too much negativity.

Providers were enthusiastic about the potential benefits of developing MBC‐specific supportive interventions. Providers viewed group‐based supportive interventions as a safe place where patients could alleviate anxiety and receive validation for their experiences. Some providers identified ways that MBC‐specific supportive interventions could benefit healthcare teams, such as re‐focusing medical appointments in which providers often end up spending time counseling patients. This point is illustrated in the following provider quote:

“It is absolutely needed… we have a 30‐minute spot with [patients]. About 10 minutes is a quick, ‘Let’s go over everything. I'm sure you’re doing well [physically].’ The rest of it is the psychosocial component and handling stress… So, I think to have an actual group that isn’t reading blogs… and have that face‐to‐face conversation, but in a peaceful setting, that would be beneficial… I think then it leaves more time [in] those clinical appointments to handle actual medical stuff.” (PV509)

Providers emphasized that any medical information shared in supportive interventions must be clinically sound. They also suggested that group‐based interventions should carefully consider how to manage social comparisons between patients and the possibility that a group member may experience disease progression or death.

Patients and providers were receptive to delivering MBC‐specific supportive interventions digitally (e.g., over videoconference) and identified possible benefits to this approach including less required travel time, increased access (e.g., for patients with children, who live far from a cancer center), and increased comfort by facilitating participation from home. The following patient quote highlights the potential benefits of digital interventions for MBC:

“Any kind of support group I've wanted to go to, I haven’t been able to because my daughter goes to pre‐school at noon or I have stuff like that… But if I could get on the computer and just do it like that, then that would make it much easier for me to be a part of.” (PT005)

Patients described feeling more confident using technology with increases in digital care due to COVID‐19. Concerns about digital interventions included potential limitations to privacy, loss of personal connections, and barriers to participation (e.g., access to required technology).

## 4 DISCUSSION

This is the first study to provide insight into the lived experiences of MBC patients taking CDK4/6 inhibitors. We explored MBC patients’ experiences with CDK4/6 inhibitor symptoms, psychosocial concerns and needs, and interest in supportive interventions using qualitative and survey‐based methods. We also qualitatively explored MBC providers’ perceptions of CDK4/6 inhibitor symptoms, observations of patients’ HRQOL concerns, and opinions about developing MBC‐specific supportive interventions.

A key theme was that perceptions of CDK4/6 inhibitor symptom burden did not align, with patients perceiving symptoms as more burdensome than providers. Patients described significant symptom burden, with fatigue in particular being both highly prevalent and burdensome. This is consistent with AE reports from clinical trials.^37^ Surveys indicated that patients’ average total symptom burden was slightly to moderately bothersome, and average fatigue scores exceeded the cutoff for severe fatigue.^33^ Of note, it may not always be possible for patients to tease apart symptoms caused by their disease versus CDK4/6 inhibitors versus other treatments such as endocrine therapy. Thus, it is important to acknowledge that patients’ reported symptoms and perceptions of symptom burden may not be attributable to CDK4/6 inhibitors alone. Nonetheless, despite their symptom burden, patients reported general HRQOL similar to normative data for diverse cancer patients and for the U.S. adult population.^38^, ^39^ In addition, patients’ breast cancer HRQOL was similar to a recent study of MBC patients taking endocrine therapy or receiving chemotherapy.^40^

Consistent with our findings, other studies show that oncology providers tend to under‐report treatment‐related symptoms by 50% or more relative to patient reports.^41^, ^42^ One explanation for varying perceptions could be different frames of reference; while providers may compare CDK4/6 inhibitor symptom burden to that of more toxic cancer treatments (e.g., cytotoxic chemotherapy), patients may not have the same experiences for comparison. As a result, providers may underestimate the impact of symptoms on patients’ HRQOL, knowing it could be worse. To bridge this gap, routinely eliciting information about patients’ symptoms and incorporating that information into clinical care may help ensure that patients’ symptoms are appropriately captured and managed. In addition, it may be just as important for oncology teams to provide patients with adequate education about their cancer treatment(s), potential symptoms, and strategies for symptom management. The use of methods such as the teach‐back technique could help to confirm patient comprehension of complex messages and improve subsequent symptom self‐management.^43^ As part of patient education and symptom management, oncology teams could consider early and consistent referrals to outpatient palliative care for patients with MBC taking CDK4/6 inhibitors given the potential for high symptom burden and indefinite treatment timeline. In such cases, communication and coordination of care between services are critical. These steps could result in improved treatment outcomes (e.g., fewer treatment dose reductions and/or discontinuations, better adherence). Finally, patients identified several reasons why they may not disclose their symptoms to their medical teams (e.g., symptoms were expected, embarrassment), which could inform strategies to encourage open and consistent patient–provider communication during and between clinical encounters. Future work may also explore how symptom disclosure and communication differ by type of provider (e.g., oncologist vs. nurse) and consider the potential implications for patients’ care.

Another key theme was that available supportive resources (e.g., support groups, blogs) did not meet patients’ needs if not specific to MBC. Patients described a disconnect when interacting with patients who have non‐metastatic disease, and they expressed a desire for resources targeted to MBC that are informative and positive. Survey data revealed that patients most frequently endorsed needing easy‐to‐understand information related to a host of topics (e.g., future condition, insurance, treatment), 40% needed better symptom control, and 20% needed to speak with their peers. These needs could all be met by developing supportive interventions that provide MBC‐relevant health information, are group‐based, and assist patients with managing the most common and distressing side effects of MBC treatments. Patients and providers were open to using digital technologies such as videoconference to deliver supportive interventions remotely, thus allowing for a group setting while increasing convenience.

Despite MBC patients’ and providers’ enthusiasm, existing interventions that address breast cancer symptom burden and HRQOL have focused almost exclusively on patients with non‐metastatic disease,^22^, ^23^, ^24^ and MBC patients are commonly excluded from supportive intervention studies. The few existing MBC supportive interventions are limited to mostly pilot studies with little evidence of efficacy, and we are unaware of any developed more recently within the context of novel life‐prolonging treatments. Patients and providers offered suggestions for developing MBC supportive interventions, which may guide future research. Suggestions included creating opportunities for peer mentorship, partnering with medical providers to ensure that health information is clinically sound, and considering how program facilitators will manage group members’ reactions to disease progression or death among their peers. Critically, the continual inclusion of MBC patients in the development of MBC supportive interventions will ensure that they are patient‐centered and adequately address patients’ lived experiences.

### 4.1 Study limitations

This study was exploratory. The small sample of MBC patients was mostly non‐Hispanic/Latina, White, highly educated, and receiving care at an NCI‐designated comprehensive cancer center. Thus, themes may not generalize to all MBC patients receiving care in community and academic oncology clinics. Future work should explore these topics in more diverse samples. In addition, this study was cross‐sectional and did not account for patients’ baseline symptoms prior to starting treatment with a CDK4/6 inhibitor nor change in symptoms over time. Future work should explore longitudinal symptom burden associated with CDK4/6 inhibitors to evaluate these factors. Finally, social constraints due to COVID‐19 at the time of study participation may have increased participants’ endorsement of group‐based and digitally delivered interventions.

### 4.2 Clinical implications

Results of this study underscore the critical need for supportive interventions specifically for MBC patients, particularly in the age of novel life‐prolonging treatments. Researchers may use these results to guide the development of MBC‐targeted supportive interventions (e.g., educational interventions, behavioral symptom management, and quality of life interventions). These results may also inform strategies to encourage open and consistent patient–provider communication during and between clinical encounters.

## 5 CONCLUSIONS

This mixed‐methods study was the first to examine the lived experiences of MBC patients taking CDK4/6 inhibitors as well as the experiences of MBC providers. Results suggest that CDK4/6 inhibitor symptoms may be more burdensome than perceived by providers, and patients expressed a need for supportive interventions specific to MBC concerns. Results can inform the development of MBC supportive interventions designed to reduce symptom burden and maintain HRQOL.

## ETHICAL APPROVAL STATEMENT

This study was reviewed by the Advarra Institutional Review Board and deemed exempt from oversight due to minimal risk (Pro00042135). All participants provided verbal informed consent to participate and to have deidentified data published in scientific reports.

## CONFLICTS OF INTEREST

Dr. Antoni is a paid consultant for Blue Note Therapeutics. Dr. Costa receives an honorarium from Bristol Meyers Squib, Pfizer, Daiichi Sankyo, Astra Zeneca, Immunomedics, and Athenex. Dr. Jim is a paid consultant for RedHill Biopharma, Janssen Scientific Affairs, and Merck. There are no other relevant conflicts of interest to disclose.

## Supporting information


**TABLE S1**. Qualitative themes and additional representative quotes

## Data Availability

The data that support the findings of this study are available from the corresponding author upon reasonable request.
