# Supplementary material for: A mixed‐methods study of cyclin‐dependent kinase 4 and 6 inhibitor symptom burden and quality of life among metastatic breast cancer patients and providers
Source: Cancer Med. 2021 Jun 24;10(14):4823–31. doi: 10.1002/cam4.4055 (PMC8290228; doi:10.1002/cam4.4055)
Supplement: Supplementary file 1 — TABLE S1. Qualitative themes and additional representative quotes [file CAM4-10-4823-s001.docx]

**Supplemental Table 1.** Qualitative themes and additional representative quotes.

| **Theme and Subtheme** | | **Representative Quote and Patient (PT) or Provider (PV) Study ID** |
| --- | --- | --- |
| **Perceptions of CDK4/6 inhibitor symptom burden did not align** | | |
| Patient perceptions of CDK4/6 inhibitor symptom burden | “I had slight fatigue with the cancer diagnosis. But… I noticed the increase in fatigue [with the CDK4/6 inhibitor] to the point where I have a lot of trouble driving... I will sleep a total of three to four hours during my day.” (PT016) | |
| Provider perceptions of CDK4/6 inhibitor symptom burden | “These agents don't really cause debilitating side effects or things that will really affect the patient's quality of life.” (PV504)  “And I think that side effects usually aren't too bad. You know, of course, when we think about a person with metastatic disease, we know that we can't cure them… And so, I think as far as tolerability goes, it's tolerated for the most part.” (PV505) | |
| Patients’ disclosure of symptoms to healthcare team | “I really don't know that there is anything they can do about [memory problems], so what's the point of saying anything? And for two, you know, it's kind of embarrassing.” (PT011) | |
| **Patients are perceived as having good HRQOL** | | |
| Patient-reported HRQOL | “I really have a renewed love of life. I think that probably comes with anyone that’s faced with this kind of diagnosis… It's made me more determined to live as long as I can and to do as much as I can.” (PT016)  “[M]y life is totally different than I ever imagined. I'd made this great business with this great social life and having my own company and everything. But I had to let all of that go.” (PT021) | |
| Provider observations of MBC patients’ HRQOL | “I'd say the biggest consequence is the quality of life issues... Because while we're trying to treat the cancer, we also know that metastatic patients living out life that they have is one of the most important things. So, we really look at that in conjunction with how we're treating them and which medications we're giving.” (PV509) | |
| **Supportive resources not specific to MBC are inadequate** | | |
| Patients’ experiences with support groups | “My best support has been a group on Facebook that are metastatic and it’s a closed group. The women are taking Ibrance and Letrozole and you can ask them anything… I was on another one, but it was women with all stages of breast cancer, so it wasn’t that helpful.” (PT001) | |
| Patient impressions of group supportive interventions targeted to MBC | “Personally, I think a group is great. But you can't have all women that are scared to death, depressed, negative, you know that kind of stuff. You have to have positive energy in there.” (PT009)  “I'm actually in an online version on Facebook for people all across the world that are on these CDK4/6 inhibitors. They're a wealth of information… You know, when you have bad days where you might crap your pants, you can go and tell them, ‘Hey, this happened to me today.’ And you can laugh about those things with them, because we're all going through the same thing. It's comfort. Where other people might see you… and they pity you. These women don't pity you because they're like, ‘Oh yeah, it happened to me.’… It's always good to talk to people that are going through the same thing that you are.” (PT017) | |
| Provider impressions of group supportive interventions targeted to MBC | “One [benefit] is a sense of community, knowing that you're not alone. And no matter how much we think we do a good job [as clinicians]… I think the reality is we don't do as good a job as we think. Or this is just such new information that people don't retain it. Or maybe they don't retain it because of the burden of disease and the medications. But it's not unusual for me to hear two or three visits later the same question, because the answer hadn't registered. And also, I think the understanding of this being kind of a long-haul thing that you don't want to push your body too much. I think it might be helpful to kind of get it from a different source.” (PV512) | |
| Perceptions of digital interventions | “I still kind of prefer some face-to-face interaction with my patients. I do think you lose a little bit of that personal touch when you're not present in a room with your peers… A lot of these women are working or they're moms and they're juggling a million things on their plate. So, to have the ease of being able to log in at home and not have to commute here and try to alter their schedules would have a lot of benefit, too.” (PV510) | |
